# CAPABLE IMPLEMENTATION: PROGRESS AND BARRIERS

**DOI:** 10.1093/geroni/igac059.1295

**Published:** 2022-12-20

**Authors:** Deborah Paone, Sarah Szanton

**Affiliations:** Johns Hopkins University, Baltimore, Maryland, United States; Johns Hopkins University School of Nursing, Baltimore, Maryland, United States

## Abstract

CAPABLE is an evidence-based 4-to-6-month program that improves daily function and the home environment based on the older adult’s goals. Study aims were to: (1) understand context and readiness factors in implementation, (2) identify barriers and facilitators, and (3) examine the utility of two frameworks. The unit of analysis was the organization. We employed the CFIR and RE-AIM frameworks for this qualitative study, using multiple data sources and traced the implementation of 40 organizations over 3 years. Leadership support, perceived value of the program, initial funding, strategic importance, and program champion were common supportive factors. Common challenges included difficulty with recruitment, staffing and infrastructure readiness and sustainability of funding. This study indicates readiness, technical support, and resources needed for implementation and sustainability of CAPABLE and suggests external environmental supports needed. It offers a practical way to monitor, evaluate, and report on ongoing implementation of evidence-based programs.

